# The alternations of nucleus accumbent in schizophrenia patients with auditory verbal hallucinations during low-frequency rTMS treatment

**DOI:** 10.3389/fpsyt.2022.971105

**Published:** 2022-09-06

**Authors:** Yuanjun Xie, Yun Cai, Muzhen Guan, Zhongheng Wang, Zhujing Ma, Peng Fang, Huaning Wang

**Affiliations:** ^1^School of Education, Xinyang College, Xinyang, China; ^2^Department of Radiology, Xijing Hospital, Air Force Medical University, Xi'an, China; ^3^Department of Neurodevelopment Psychology, School of Psychology, Army Medical University, Chongqing, China; ^4^Department of Mental Health, Xi'an Medical University, Xi'an, China; ^5^Department of Psychiatry, Xijing Hospital, Air Force Medical University, Xi'an, China; ^6^Department of Clinical Psychology, Air Force Medical University, Xi'an, China; ^7^Department of Military Medical Psychology, Air Force Medical University, Xi'an, China

**Keywords:** schizophrenia, auditory verbal hallucination, nucleus accumbent, functional connectivity, gray matter volume, repetitive transcranial magnetic stimulation

## Abstract

Low-frequency repetitive transcranial magnetic stimulation (rTMS) has been shown to reduce the severity of auditory verbal hallucinations (AVH) and induce beneficial functional and structural alternations of the brain in schizophrenia patients with AVH. The nucleus accumbens (NAcc) as an important component of the ventral striatum is implicated with the pathology in AVH. However, the induced characteristic patterns of NAcc by low-frequency rTMS in schizophrenia with AVH are seldom explored. We investigated the functional and structural characteristic patterns of NAcc by using seed-based functional connectivity (FC) analysis and gray matter volume (GMV) measurement in schizophrenia patients with AVH during 1 Hz rTMS treatment. Although low-frequency rTMS treatment did not affect the volumetric changes of NAcc, the abnormal FC patterns of NAcc, including increased FC of NAcc with the temporal lobes and decreased FC of NAcc with the frontal cortices in the pretreatment patients compared to healthy controls, were normalized or reversed after treatment. These FC changes were associated with improvements in clinical symptoms and neurocognitive functions. Our findings may extend our understanding of the NAcc in the pathology of schizophrenia with AVH and might be a biomarker of clinical effect for low-frequency rTMS treatment in schizophrenia.

## Introduction

Schizophrenia is a chronic and disabling disease that affects ~0.7% of the population (1). Symptoms associated with schizophrenia can be divided into three domains: positive symptoms (e.g., hallucinations and delusions), negative symptoms (e.g., avolition and withdrawal), and cognitive symptoms (e.g., memory and executive function) (2). The etiology of schizophrenia is still poorly understood. However, the neurobiology of the psychotic symptoms has been associated with dopaminergic abnormality in the striatum (3). Abnormal dopaminergic regulation of striatal function could explain the mechanisms underlying the symptoms of schizophrenia (4, 5). Of particular interest is the nucleus accumbens (NAcc), a central component of the ventral striatum, which plays an important role in the pathology of schizophrenia (6). The modulation of the striatal circuit activity can reduce psychotic symptoms (7). Thus, NAcc has been proposed as the critical target for antipsychotic medications (8).

NAcc receives intensive excitatory afferents from the frontal cortex, hippocampus, and amygdala, closely associated with dopaminergic changes in schizophrenia pathology (9). Several studies have reported increased dopaminergic activity in the NAcc in schizophrenia (10, 11). Subsequent animal studies have confirmed similar findings (12–14). Structural abnormalities in the NAcc have been consistently illustrated in schizophrenia. There were significant reductions in gray matter volume (GMV)of the NAcc in schizophrenic brains from the structural magnetic resonance imaging data (15–17). In addition, resting-state functional magnetic resonance imaging (fMRI) studies have observed abnormal intrinsic functional connectivity (FC) of NAcc in schizophrenia (18–20), regions mainly located in the frontal, parietal, temporal, and limbic systems (e.g., the cingulate cortex, insula, parahippocampal gyrus, and ventral tegmental area). Therefore, NAcc is the primary region interacting with multiple areas of cortical and limbic systems and could provide a supplementary understanding of pathology in schizophrenia.

Current treatments of antipsychotics are thought to target the NAcc and can reduce a hyperdopaminergic state of the striatum (21, 22). However, antipsychotics are only responded to symptoms and are confined in their effectiveness, and frequently accompanied by side effects (23). Meta-analysis and system review studies have indicated that the application of low-frequency repetitive transcranial magnetic stimulation (rTMS) during schizophrenia can effectively reduce the severity of auditory verbal hallucinations (AVH) (24–28), although negative findings were reported (29, 30), probably because the heterogeneity of treatment protocols and placebo response (31). AVH are defined as perceptions in the absence of external verbal stimuli and are prominent among the core symptoms of schizophrenia (32). The activation of NAcc is associated with the vividness of hallucinations (33) and auditory verbal imagery in schizophrenia patients (34). Moreover, the abnormal FC (20, 35) and gray matter changes (36) of NAcc appeared to be associated with the presence of AVH and neurocognitive impairments. The results may indicate the unique role of NAcc in investigating the neural mechanisms of schizophrenia with AVH. Nevertheless, its underlying changes in schizophrenia with AVH during rTMS are seldom explored.

The purpose of the present study aimed to investigate the potential alternations of NAcc in schizophrenia patients with AVH during low-frequency rTMS treatment by using the seed-based FC analysis and GMV measurement. Correlation analyses were further done between the possible alternations of NAcc and clinical responses of patients after treatment. We hypothesized that low-frequency rTMS treatment could normalize or inverse the abnormal functional or structural patterns of NAcc and associated with the reduction of clinical symptom severity.

## Materials and methods

### Participants

Thirty-two patients with AVH were recruited from the Department of Psychiatry, Xijing Hospital of Fourth Military Medical University. The diagnosis of schizophrenia was made by experienced psychiatrists according to the Chinese version of the Structured Clinical Interview for Diagnosis and Statistical Manual of Mental Disorder (DSM-V). The inclusion criteria of the patient group were as follows: (1) AVH daily occurred with at least two antipsychotic medications, and (2) no less than five episodes of AVH per day over the past month. All patients who received a steady dose of antipsychotic medications remained unchanged during the study period. In addition, thirty-five healthy controls matched by age, sex, and education were recruited from the local community through advertising and had no history of psychiatric diseases. For all the participants, the exclusion criteria were as follows: (1) any past or current neurological diseases, (2) history of head injury, (3) alcohol or substance abuse, and (4) contraindications to MRI scans.

This study was approved by the Medical Ethics Committee of the Xijing Hospital and was conducted following the Declaration of Helsinki. Informed written consent was obtained from all the participants. The study was registered in the Chinese Clinical Trial Register (http://www.chictr.org/cn/, registration number: ChiCTR2100041876).

### Clinical measurements

The severity of psychotic symptoms was assessed using the Positive and Negative Syndrome Scale (PANSS) (37). The AVH was assessed by the auditory Hallucination Rating Scale (AHRS) (38). The Chinese version of the MATRICS Consensus Cognitive Battery (MCCB) was used to measure neurocognitive impairment in patients consisting of 10 tasks across seven cognitive domains (39): speed of processing test (SOPT), attention and vigilance test (AVT), working memory (WMT) test, verbal learning test (VERBLT), visual learning test (VISLT), reasoning and problem-solving test (RPST), and social cognition test (SCT). All clinical measures were performed by experienced psychiatrists at baseline and after treatment.

### rTMS protocol

A type of 8-figure coil magnetic stimulator (YIRUIDE Inc., Wuhan, China) was used to perform 1 Hz rTMS treatment, and the left temporoparietal junction (TPJ) was selected as the stimulation target, which is referred to as the International 10–20 electrode location system (TP3). This stimulation target has been widely applied to treat AVH in schizophrenia by using low-frequency rTMS (40, 41). Patients were treated for 15 consecutive days at 15 min per day (once per second, 5 s interval) with a 110% resting motor threshold, generating 60 trains of 600 pulses.

### MRI data acquisition

MRI data were obtained using a 3.0-Tesla scanner (GE Medical Systems, Milwaukee, WI) equipped with an 8-channel phased-array head coil. The patient group was scanned twice (before and after treatment), while the control group was scanned only once. During the entire scan, the participants were instructed to stay awake with their eyes closed and remain awake and keep their heads motionless. Resting-state functional images were obtained using a gradient-echo-planar imaging sequence with the following parameters: 45 axial slices, repetition time (TR) = 2,000 ms, echo time (TE) = 40 ms, matrix = 64 × 64, field of view (FOV) = 260 × 260 mm^2^, flip angle = 90°; slice thickness = 3.5 mm (no gap), and 210 volumes were acquired. The T1-weighted structural images were obtained during the same scanning session by an MP-RAGE sequence as the following parameters: TR = 8.1 ms, TE = 3.2 ms, matrix size = 256 × 256, flip angle = 12°, FOV = 240 × 240 mm^2^, 176 slices, and thickness=1.0 mm.

### Neuroimaging data preprocessing

Resting-state functional imaging data were preprocessed using the SPM (https://www.fil.ion.ucl.ac.uk/spm/) and DPABI (http://rfmri.org/dpabi) toolbox. For each participant, the first ten functional volumes were removed to assure equilibration of the magnetic field. The remaining volumes were corrected for slice acquisition and head motion. Subsequently, the corrected images were normalized into the standard Montreal Neurological Institute (MNI) space by the Exponentiated Lie Algebra (DARTEL) algorithm (42) and then resampled to a 3 × 3 × 3 mm^3^ resolution. Then, the normalized images were linearly detrended and regressed the nuisance covariates, including Friston 24 motion parameters (43), white matter signal, cerebrospinal fluid signal, and whole-brain global signal. Band-pass temporal filtering (0.01–0.1 Hz) was performed to reduce high-frequency physiological noise. Finally, spatial smoothing was conducted with a 6-mm Gaussian kernel for statistical analyses.

Structural imaging data were processed using SPM (https://www.fil.ion.ucl.ac.uk/spm/) and VBM (https://dbm.neuro.uni-jena.de/wordpress/vbm/) toolbox. The structural images were subjected to bias correction and tissue-classified into gray matter, white matter, and cerebrospinal fluid with the volume probability maps. The gray matter images were then normalized to standard Montreal Neurological Institute (MNI) space. Subsequently, intensity modulation and an 8 mm Gaussian kernel smoothing of the resulting images were completed.

### FC analysis

**Figure 1:**
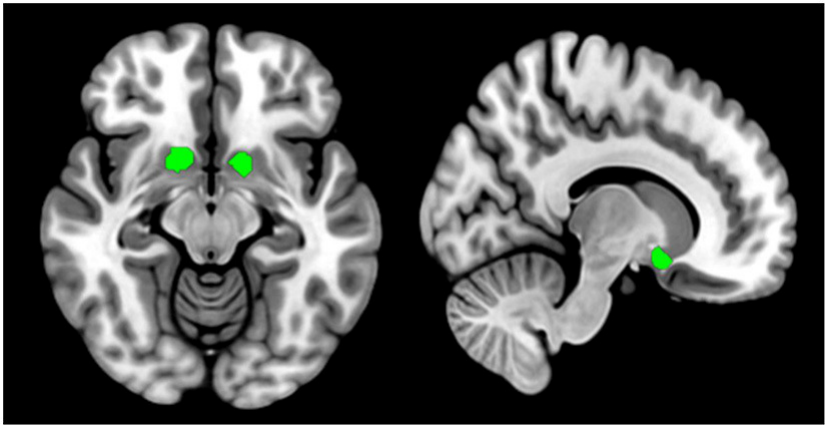
Nucleus accumbent seed regions of interest are defined by the Anatomical Automatic Labeling (AAL3) atlas (https://www.oxcns.org/aal3.html). Bilateral nucleus accumbent seeds are used in resting-state functional connectivity analysis and gray matter volume measure.

The bilateral NAcc were defined as seeds based on the Anatomical Automatic Labeling (AAL3) atlas (44), see Figure 1 for details. Subsequent procedures were executed in the left and right seed individually. Pearson correlation analyses were performed between the seed reference time course and time series of the whole brain. The resulting correlation coefficients were converted into z-scores using to enhance normality.

### GMV analysis

The values of GMV from the NAcc were then extracted from the preprocessed gray matter images with the seed mask. The GMV differences of left and right NAcc were then compared between the patient and control groups or patients before and after treatment.

### Statistical analysis

Statistical analysis of the demographic and clinical data was carried out using the SPSS (version 23.0; Chicago, IL, United States). Independent-sample *t*-test and chi-square test were conducted according to the characteristics of the data. In addition, the independent-sample *t*-tests were done to investigate group differences in FC and GMV between patients at baseline and controls with age, gender, education, and mean head motion (Framewise displacement, FD) parameter as covariates. These different brain regions were defined as a mask for subsequent analysis. A paired-sample *t*-test was used to examine the treatment effect of the two measures between patients after treatment and before treatment with the mask created above. Group statistical maps were thresholded at *p* < 0.05 and a voxel level of *p* < 0.05 with 30 voxel size using the Gaussian random field (GRF) method.

Finally, partial correlation coefficients were calculated between the altered measures and clinical responses in patients using the dosage of antipsychotics as a covariate. To explore the effect of antipsychotics on clinical symptoms and measure changes, correlations of the medication dosage with clinical response and measure changes were examined. For all correlation coefficients, a two-tailed p level of 0.05 was used as the criterion of statistical significance and corrected for multiple comparisons with the false discovery rate correction (FDR) method.

## Results

### Demographic and clinical data comparisons

The demographic and clinical characteristics of the participants are displayed in Table 1. The difference in age (*t* = 0.954, *p* = 0.345), sex (χ^2^ = 0.101, *p* = 0.751), and educational years (*t* = 1.708, *p* = 0.094) distribution did not reach significance in the patients at baseline and controls.

**Table 1:** Demographic and clinical characteristics of the participants.

**Variable**	**Patients (*****n*** = **30)**	**Controls (*****n*** = **33)**	**χ^2^ (*t*)**	***p*** **-value**
Age (year)	30.30 ± 4.46	32.03 ± 7.31	0.954	0.345
Sex (female/male)	17 (13)	20 (13)	0.101	0.751
Education (year)	13.20 ± 2.67	12.09 ± 2.04	1.708	0.094
Duration of illness (month)	21.36 ± 4.89	–	–	–
Medication dosage (CPED, mg/day)	584.8 ± 152.39	–	–	–
Medication duration (day)	15	–	–	–

CPED, Chlorpromazine equivalent doses (45).

But after rTMS treatment, the clinical responses, including positive symptoms (14.45 ± 2.80 vs. 19.65 ± 4.60, *t* = 4.324, *p* = 0.000), AVH (13.75 ± 7.07 vs. 27.45 ± 6.14, *t* = 6.542, *p* = 0.000), and certain neurocognitive functions such as verbal memory (29.60 ± 12.60 vs. 39.80 ± 12.24, *t* = 2.597, *p* = 0.047) and visual memory (34.55 ± 15.95 vs. 47.00 ± 10.54, *t* = 2.912, *p* = 0.042), were improved in patients compared to before treatment. Details are displayed in Table 2.

**Table 2:** Comparisons of clinical responses between patients before and after treatment.

	**Pretreatment (*n* = 30)**	**Posttreatment (*n* = 30)**	***t***	***p*-value**
**PNASS**				
Total scores	79.85 ± 10.55	67.50 ± 7.98	4.175	0.000
Positive symptoms	19.65 ± 4.60	14.45 ± 2.80	4.324	0.000
Negative symptoms	19.85 ± 4.53	17.85 ± 2.96	1.652	0.107
General symptoms	40.35 ± 6.65	35.20 ± 5.54	2.661	0.011
**AHRS**	27.45 ± 6.14	13.75 ± 7.07	6.542	0.000
**MCCB**				
SOPT	27.20 ± 14.61	34.15 ± 10.96	1.702	0.137
AVT	35.80 ± 13.00	42.20 ± 8.35	1.852	0.127
WMT	32.95 ± 12.34	39.75 ± 14.37	1.606	0.137
VERBLT	29.60 ± 12.60	39.80 ± 12.24	2.597	0.047
VISLT	34.55 ± 15.95	47.00 ± 10.54	2.912	0.042
RPST	35.45 ± 13.63	43.85 ± 12.38	2.040	0.114
SCT	31.95 ± 6.72	34.20 ± 7.49	1.000	0.327

PNASS, positive and negative syndrome scale; AHRS, auditory hallucination rating scale; MCCB, MATRICS Consensus Cognitive Battery; SOPT, speed of processing test attention; AVT, vigilance test; WMT, working memory test; VERBLT, verbal learning test; VISLT, visual learning test; RPST, reasoning and problem-solving test; SCT, social cognition test.

### FC comparison of NAcc seeds

**Figure 2:**
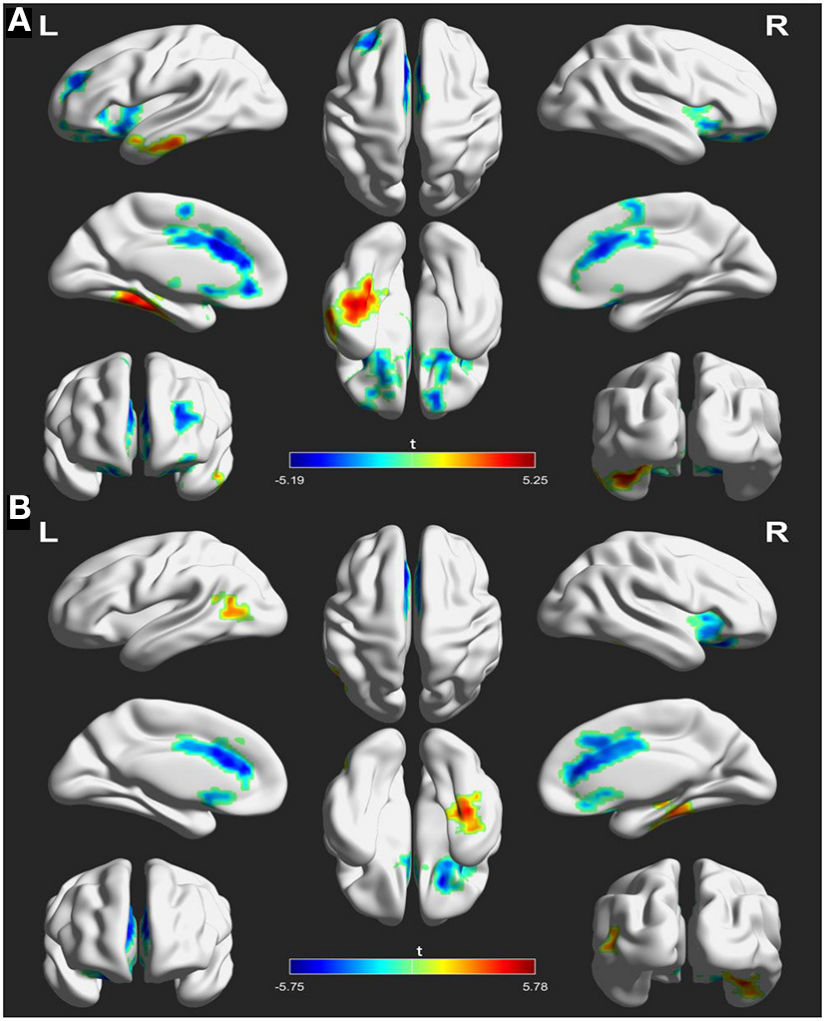
Differences in functional connectivity (FC) between patients at baseline and healthy controls using left nucleus accumbent **(A)** and right nucleus accumbent **(B)** seed regions. The warm color indicates an increased FC of seed with the whole brain and the cool color indicates a decreased FC of seed with the whole brain. The color scale is represented by the *t*-value of statistically significant clusters with the voxel-level statistical threshold of *p* < 0.05 and a cluster-level threshold of *p* < 0.05 corrected for the Gaussian random field (size >30).

The analyses of FC in the NAcc seeds between patients at baseline and controls are shown in Figure 2 and Table 3. For the left NAcc seed, the patients exhibited significantly increased FC in the left inferior temporal gyrus and right fusiform gyrus, and decreased FC in the right superior frontal gyrus and left anterior cingulate gyrus when compared with the controls (GRF correction; voxel-level *p* < 0.05, cluster level *p* < 0.05, clusters size > 30 voxels). Similar, significantly increased FC of the right NAcc seed was seen in the left middle temporal gyrus and right fusiform gyrus, and decreased FC was seen in the right inferior frontal gyrus and left anterior cingulate gyrus in patients at baseline relative to the controls (GRF correction; voxel-level *p* < 0.05, cluster level *p* < 0.05, clusters size > 30 voxels). These abnormal FC regions were defined as mask for subsequent comparisons between patients before and after treatment.

**Table 3:** Functional connectivity differences of the nucleus accumbens seeds between patients at baseline and controls (patients > controls).

**Brain regions**	**Side**	**BA**	**Cluster size**	**MNI coordinates**			***t*-value**
				***x***	***y***	***z***	
**L NAcc seed**							
Inferior temporal gyrus	L	37	242	−39	−33	−18	5.250
Fusiform gyrus	R	30	83	24	−30	−21	4.162
Superior frontal gyrus	R	48	77	24	12	−15	−4.797
Anterior cingulate gyrus	L	25	197	−9	27	18	−5.193
**R NAcc seed**							
Fusiform gyrus	R	20	95	36	−30	−18	5.776
Middle temporal gyrus	L	37	132	−54	−69	9	3.832
Inferior frontal gyrus	R	11	43	21	27	−21	−4.911
Anterior cingulate gyrus	L	24	188	−9	33	15	−5.750

NAcc, nucleus accumbens; L, left; R, right; BA, Brodmann area; MNI, Montreal Neurological Institute.

**Figure 3:**
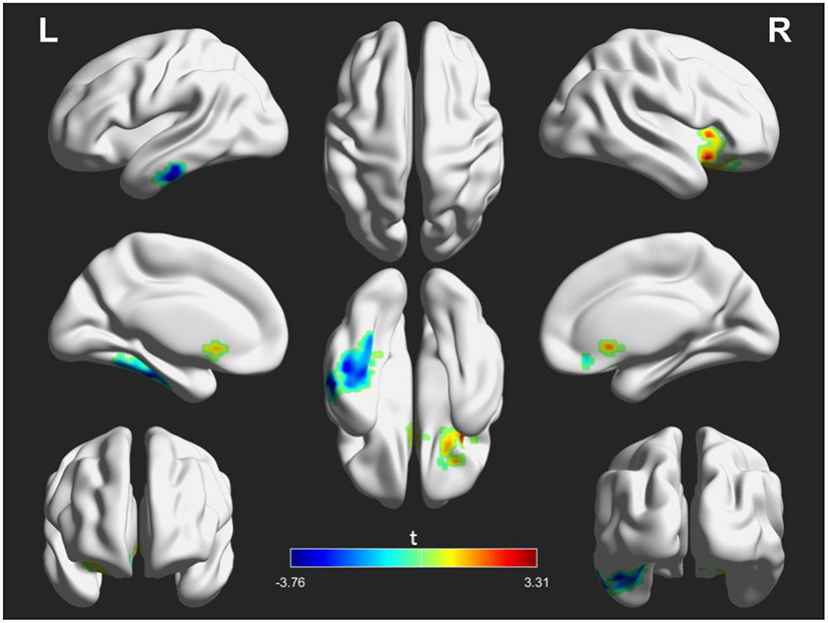
Differences in functional connectivity (FC) of the nucleus accumbens seeds between patients after treatment and before treatment. The warm color indicates an increased FC of seed with the whole brain and the cool color indicates a decreased FC of seed with the whole brain. The color scale is represented by the *t*-value of statistically significant clusters with the voxel-level statistical threshold of *p* < 0.05 and a cluster-level threshold of *p* < 0.05 corrected for the Gaussian random field (size >30).

However, these abnormal FC patterns did not persistent after rTMS treatment. Instead, initial FC of NACC with the left inferior temporal gyrus (posttreatment vs. pretreatment: −0.025 ± 0.089 vs. 0.023 ± 0.097, *t* = 2.723, *p* = 0.011) and right inferior frontal gyrus (posttreatment vs. pretreatment: 0.235 ± 0.108 vs. 0.180 ± 0.122, *t* = 2.652, *p* = 0.013) in patients before treatment was inversed after treatment. Details are displayed in Figure 3 and Table 4.

**Table 4:** Functional connectivity differences of the nucleus accumbens seeds between patients after treatment and before treatment (after treatment > before treatment).

**Brain regions**	**Side**	**BA**	**Cluster size**	**MNI coordinates**			***t*-value**
				***x***	***y***	***z***	
**L NAcc seed**							
Inferior temporal gyrus	L	20	90	−57	−18	−30	−3.759
**R NAcc seed**							
Inferior frontal gyrus	R	48	32	33	23	−7	3.314

NAcc, nucleus accumbens; L, left; R, right; MNI, Montreal Neurological Institute.

### GMV comparison of NAcc seeds

**Figure 4:**
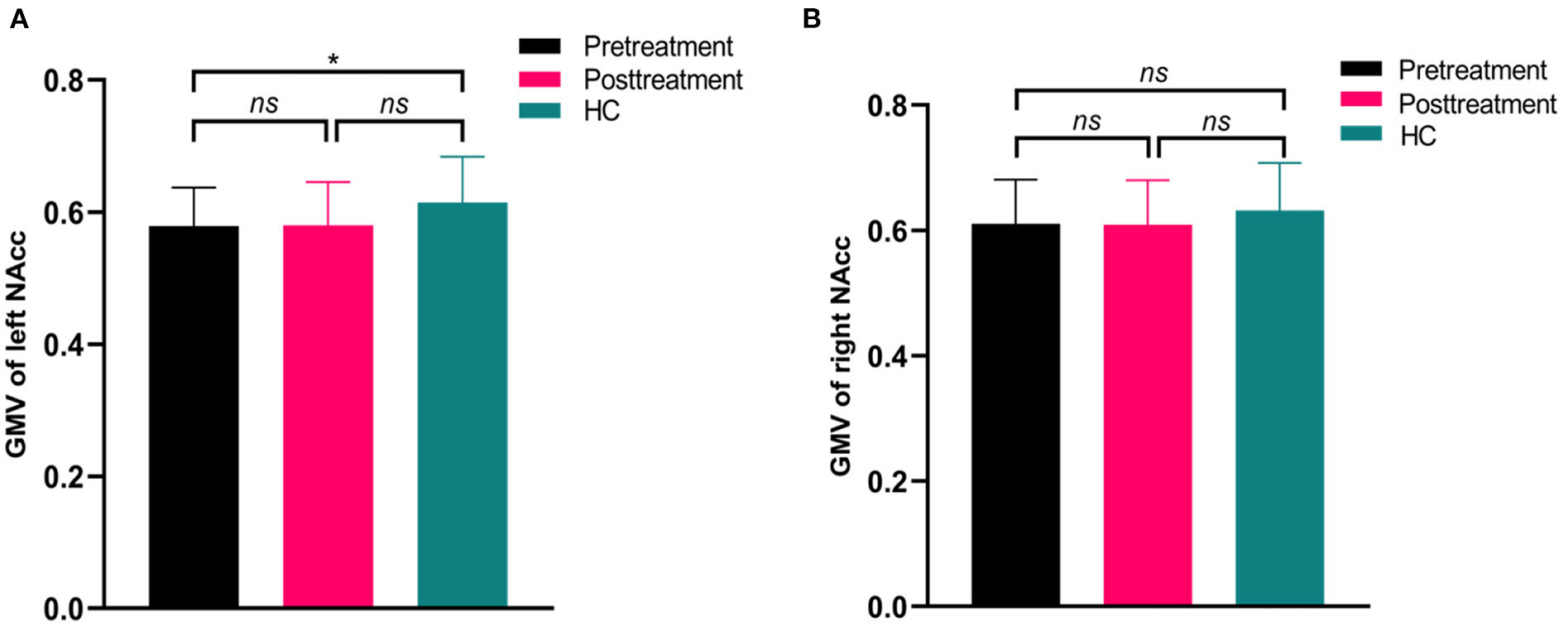
Differences in gray matter volume (GMV) of nucleus accumbens between patients (pretreatment and posttreatment) and healthy controls (HC). The pretreatment patients showed significantly decreased GMV in the left nucleus accumbent compared to HC **(A)**. While there were no significant differences in the right nucleus accumbens between patients (pretreatment and posttreatment) and HC **(B)**. *p > 0.05; ns, no significance.

The volumetric analysis showed that the patients at baseline had decreased GMV in left NAcc compared to the controls (*t* = 2.18, *p* = 0.038) (Figure 4), while the rTMS treatment did not affect the volumetric changes in the left or right NAcc in patients (*p* > 0.05) (Figure 4).

### Correlation analysis

**Figure 5:**
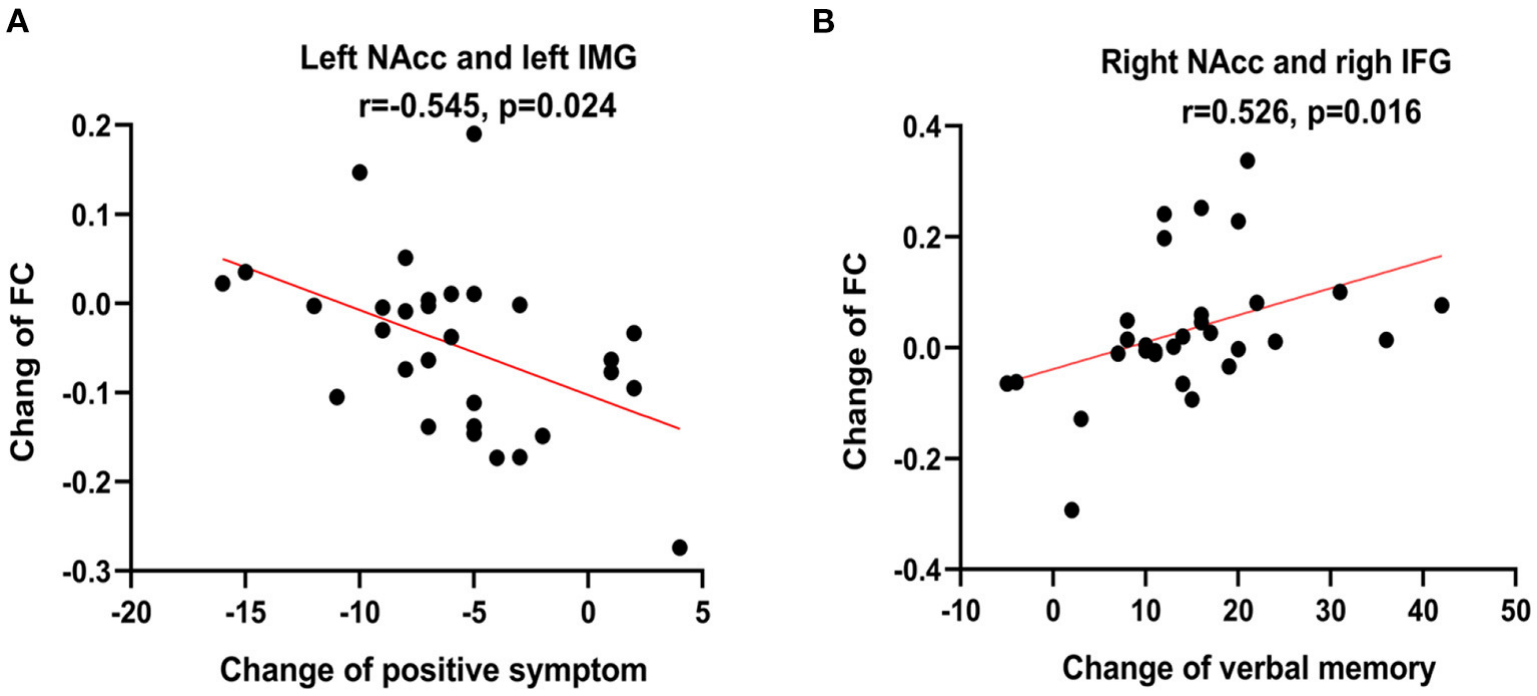
Correlations analysis showed that the changed functional connectivity (FC) value of left nucleus accumbens (NAcc) with the left inferior temporal gyrus (IMG) is negatively correlated with the change of positive symptom score of PNASS (*r* = −0.545, *p* = 0.024, False discovery rate correction) **(A)** and FC value of the right NAcc with the right inferior frontal gyrus (IFG) is positively correlated with the change of verbal memory score (*r* = 0.526, *p* = 0.016, False discovery rate correction) **(B)**.

In the patient group, the changed FC value in the left NAcc seed with the left inferior temporal gyrus was positively correlated to the changed positive symptom score of PNASS (*r* = −0.545, *p* = 0.024, FDR correction). In addition, the changed FC value of the right NAcc seed with the right inferior frontal gyrus was negatively correlated to changed verbal memory score (*r* = 0.526, *p* = 0.016, FDR correction) in the patients. But the medication dosage was not significantly correlated with the clinical symptom score FC value changes (all *p* > 0.05, Supplementary Table 1). Details are displayed in Figure 5.

## Discussion

In the present study, we investigated FC and GMV alternations of NAcc when schizophrenia patients with AVH received low-frequency rTMS treatment. Our findings demonstrated the patients at baseline had abnormal FC of NAcc with the temporal, frontal, and anterior cingulate cortices and decreased GMV in left NACC compared to controls. Although low-frequency rTMS did not affect the volumetric changes of NAcc, the abnormal FC patterns of NAcc with the temporal and frontal cortices were reversed in patients after treatment. The alternations of FC patterns were associated with clinical improvements in patients. These findings suggested that the NAcc may play an important role in the underlying pathology of schizophrenia and contribute to the effect of low-frequency rTMS on schizophrenia patients with AVH.

Our results indicated that patients at baseline had higher FC of NAcc with the temporal lobes (left middle temporal gyrus, left inferior temporal, and right fusiform gyrus) compared to control. These regions represent the speech processing areas (46, 47) and are known to be associated with AVH (48). Neuroimaging studies have indicated that auditory hallucinations are associated with hyperactivity in the auditory language cortex (49–51). Increased metabolism of temporal lobes has been reported in schizophrenia patients (52) and was related to positive symptoms (53). The hyperactive FC between NAcc and temporal lobes might be involved in an impaired function in speech perceptions and could be associated with the poor functional outcomes of patients with AVH. Higher FC between the NAcc and the temporal lobes appearing in schizophrenia patients with AVH was agreed with the previous report (19, 20), which might suggest a functional deficit of langue processing in the striatum-related circuits.

Decreased FC of NAcc with the frontal cortices (e.g., right superior frontal gyrus and inferior frontal gyrus) and anterior cingulate gyrus was also observed in patients at baseline relative to controls. The results are consistent with previous studies that reported hypoconnectivity of the frontostriatal loop in schizophrenia (18, 54). Specifically, Broca's region and its right hemisphere counterpart in the inferior frontal gyrus are involved in language processing (55, 56). There is common activation between the inferior frontal gyrus and NAcc during cognitive task processing (57) and decreased tract connections between them in schizophrenia (58, 59). In addition, the anterior cingulate gyrus is a critical area to integrate cognitive control processes (e.g., error monitoring) (60–62). Dysfunction of this region is found in schizophrenia (63) and may involve the misattribution of external sources of speech (64). The decreased FC of NAcc may partly explain the cognitive control deficits in patients that are characteristic of the clinical manifestations of schizophrenia, since the NAcc is implicated in cognitive functions, including memory, motivation, and decision-making (65) and is a virtual interface for information transmission between cortical and subcortical structures (66). Therefore, this hypoconnectivity of the NAcc circuit may lead to impairments of langue processing in schizophrenia.

However, these abnormal FC patterns of NAcc were normalized or inversed in patients after rTMS treatment. Several studies have indicated that low-frequency rTMS can increase the contribution of connected regions associated with auditory hallucinations (40, 41) due to long-lasting neuroplastic changes derived from the rTMS. Thus, the clinical effect of low-frequency rTMS on AVH may be associated with the reduction of hyperactivity in the auditory language cortex and relevant areas that propagate through remote pathways. Consistent with the hypothesis, we observed that initial increased FC between the NAcc and left inferior temporal gyrus in patients were inversed after treatment, which supports the long-term depression phenomenon induced by low-frequency rTMS (67). The inhibitory effect may shift from the target site to adjacent regions (e.g., NAcc) since there are well-established projections between them (68). This beneficial alternation may lead to the induced spread of the physiological effect in the auditory language circuit and may play an indirect modulatory effect on the NAcc connection loops, which could be associated with the reduction of clinical symptoms (e.g., positive symptom).

In addition, we found that the decreased FC of NAcc with the right inferior frontal gyrus was reversed in patients after rTMS treatment. Induced metabolic alternation in the frontal cortex by the low-frequency rTMS has been reported in schizophrenia with AVH (52). This alternation could be due to the induction of integration of frontotemporal disconnection that is documented in schizophrenia (69, 70). Studies have indicated that the NAcc connected with the inferior frontal gyrus (20) and TMS over the frontal cortex can induce dopamine and glutamate changes in the NAcc (71). These findings suggest that low-frequency rTMS could have a modulatory effect on neurotransmitters released in the NAcc through the remote effects of stimulation at the interconnected regions and thus could be associated the neurocognitive improvements such as verbal learning and memory.

Structural abnormalities in NAcc have been consistently demonstrated in schizophrenia. Two meta-analyses studies showed significant reductions in NAcc volume in patients with schizophrenia (16, 72). There is evidence from studies in adolescents (73) and adults (74) that NACC volumes are larger in the left but not in the right hemisphere. However, we found a significantly smaller volume in the left NACC in patients compared to controls. This finding was consistent with the previous studies that deficit schizophrenia patients displayed smaller left NAcc volumes compared to controls (75) and may reflect the changes in structural asymmetries in the schizophrenia brain. Although we did not find any volumetric changes in NAcc in patients after rTMS treatment, the asymmetry of NAcc presented in schizophrenia may represent the alternations in specific deep gray matter nuclei associated with an endophenotype of schizophrenia with AVH.

Some limitations of the present stud should be considered. Firstly, the sample size was small and limited the statistical power. Future studies should consider collecting larger datasets to improve the statistical power. Secondly, patients enrolled in this study were under stable antipsychotic medication treatment, and the impact of antipsychotic medication on FC of the NAcc should be taken into account, although no correlations were found between medication and FC alterations of NAcc in patients. Finally, the absence of placebo sham stimuli may lead to caution about the efficacy of the stimulus paradigm.

## Conclusions

In summary, our findings revealed abnormal FC and GMV changes of NAcc in patients and suggested an involvement of the striatal pathway in schizophrenia with AVH. Moreover, the abnormal FC patterns of the NAcc were inversed by low-frequency rTMS treatment and could be biomarkers of the clinical effectiveness of low-frequency rTMS treatment in schizophrenia with AVH.

## Data availability statement

The original contributions presented in the study are included in the article/Supplementary material, further inquiries can be directed to the corresponding author/s.

## Ethics statement

The studies involving human participants were reviewed and approved by the Medical Ethics Committee of the Xijing Hospital. The patients/participants provided their written informed consent to participate in this study.

## Author contributions

YX, MG, and ZW design and organized the research. YX, YC, ZW, and PF collected the imaging and cognitive data. YX and PF analyzed the data. YX, YC, and MG wrote and revised the manuscript. PF and HW provided fund support. All authors contributed to the article and approved the submitted version.

## Funding

This study was funded by the Natural Science Foundation of China (Nos. 81971255 and 81571651), Postdoctoral Science Foundation (2019M653963), and the Military Medical Science and Technology Youth Training Program of China (20QNPY049).

## Conflict of interest

The authors declare that the research was conducted in the absence of any commercial or financial relationships that could be construed as a potential conflict of interest.

## Publisher's note

All claims expressed in this article are solely those of the authors and do not necessarily represent those of their affiliated organizations, or those of the publisher, the editors and the reviewers. Any product that may be evaluated in this article, or claim that may be made by its manufacturer, is not guaranteed or endorsed by the publisher.
