# Corrigendum: The alternations of nucleus accumbent in schizophrenia patients with auditory verbal hallucinations during low-frequency rTMS treatment

**DOI:** 10.3389/fpsyt.2022.1118275

**Published:** 2023-01-04

**Authors:** Yuanjun Xie, Yun Cai, Muzhen Guan, Zhongheng Wang, Zhujing Ma, Peng Fang, Huaning Wang

**Affiliations:** ^1^School of Education, Xinyang College, Xinyang, China; ^2^Department of Radiology, Xijing Hospital, Air Force Medical University, Xi'an, China; ^3^Department of Neurodevelopment Psychology, School of Psychology, Army Medical University, Chongqing, China; ^4^Department of Mental Health, Xi'an Medical University, Xi'an, China; ^5^Department of Psychiatry, Xijing Hospital, Air Force Medical University, Xi'an, China; ^6^Department of Clinical Psychology, Air Force Medical University, Xi'an, China; ^7^Department of Military Medical Psychology, Air Force Medical University, Xi'an, China

**Keywords:** schizophrenia, auditory verbal hallucination, nucleus accumbent, functional connectivity, gray matter volume, repetitive transcranial magnetic stimulation

In the published article, there was an error in Table 1 as published. The time measurement of the fourth variable (fourth line, first column) was incorrect. The corrected Table 1 and its caption appear below.

**Table 1:** Demographic and clinical characteristics of the participants.

**Variable**	**Patients (*n* = 30)**	**Controls (*n* = 33)**	**χ^2^(t)**	***p*-value**
Age (year)	30.30 ± 4.46	32.03 ± 7.31	0.954	0.345
Sex (female/male)	17 (13)	20 (13)	0.101	0.751
Education (year)	13.20 ± 2.67	12.09 ± 2.04	1.708	0.094
Duration of illness (month)	21.36 ± 4.89	–	–	–
Medication dosage (CPED, mg/day)	584.8 ± 152.39	–	–	–
Medication duration (day)	15	–	–	–

CPED, Chlorpromazine equivalent doses (45).

The authors apologize for this error and state that this does not change the scientific conclusions of the article in any way. The original article has been updated.

