# Supplementary material for: The alternations of nucleus accumbent in schizophrenia patients with auditory verbal hallucinations during low-frequency rTMS treatment
Source: Front Psychiatry. 2022 Sep 6;13:971105. doi: 10.3389/fpsyt.2022.971105 (PMC9485869; doi:10.3389/fpsyt.2022.971105)
Supplement: Supplementary file 1 [file Table_1.docx]

**Supplementary Table 1** Correlations of medication information and changed clinical responses and measures in patients

|  | Medication Dosage |
| --- | --- |
| **Functional Connectivity Change** |  |
| L_NAcc – L_ITG | -0.241 (0.199) |
| R_NAcc – R_IFG | -0.214 (0.366) |
| **Clinical Response Change** |  |
| Positive Symptom of PNASS | -0.230 (0.222) |
| General Symptom of PNASS | 0.071 (0.711) |
| Total Score of PNASS | -0.156 (0.409) |
| AHRS | -0.111 (0.558) |
| Verbal Memory | 0.234 (0.213) |
| Visual Memory | 0.184 (0.329) |

Note: L, left; R, right; ITG, inferior temporal gyrus; IFG, inferior frontal gyrus; NAcc, nucleus accumbens; PANSS, positive and negative symptom scale; AHRS, auditory hallucination rating scale. Number in parentheses indicate a significance level.
